# Correlation of changes in circulating levels of hypoxia-induced factor-1α, erythropoietin, and cell-free fetal hemoglobin with feto-maternal pregnancy outcomes

**DOI:** 10.1590/1414-431X2025e14354

**Published:** 2025-10-06

**Authors:** A.A. Mohamed, I.A. Albahlol, E.A. Alzarea, A.B.A. Alshaikh, F.E. Edris, M.S. Abdel-Tawab, T.H. El-Metwally

**Affiliations:** 1 Biochemistry Division, Department of Pathology, College of Medicine, Jouf University, Sakaka, Saudi Arabia; 2 Department of Obstetrics and Gynecology, College of Medicine, Jouf University, Sakaka, Saudi Arabia; 3 Department of Obstetrics and Gynecology, Faculty of Medicine, Mansoura University, Mansoura, Egypt; 4 Hematopathology, Department of Pathology, College of Medicine, Jouf University, Sakaka, Saudi Arabia; 5 Department of Obstetrics and Gynecology, College of Medicine, Umm Al-Qura University, Makkah, Saudi Arabia; 6 Department of Medical Biochemistry, Faculty of Medicine, Beni-Suef University, Beni-Suef, Egypt; 7 Department of Medical Biochemistry and Molecular Biology, Faculty of Medicine, Assiut University, Assiut, Egypt

**Keywords:** Pregnancy, Feto-maternal pregnancy outcomes, Hypoxia biomarkers, Erythropoietin, Hypoxia-induced factor-1α, Cell-free hemoglobin F

## Abstract

Pregnancy feto-maternal complications (PFMCs) contribute significantly to morbidity and mortality, with placental hypoxia as a key factor. Current methods for detecting fetal hypoxia are limited. This study aimed to evaluate the roles and biomarker potential of hypoxia-inducible factor (HIF)-1α, erythropoietin (EPO), and cell-free fetal hemoglobin (cf-HbF) in PFMCs. In a cross-sectional study, we quantitatively immunoassayed plasma levels of these biomarkers in 136 healthy pregnant women (age 29.618±5.19 years) and 118 women with complications (age 30.53±5.46 years) who had voluntarily consented and were enrolled sequentially and anonymously. Results indicated significantly higher biomarker levels in complicated pregnancies (P<0.001), with worsening trends for preeclampsia, ectopic pregnancy, abortion, gestational diabetes, and preterm labor with premature rupture of membranes. Only EPO levels showed pregnancy duration-dependent changes in healthy controls (encompassing 6 to 41 weeks; r=0.230, P=0.015). Strong correlations among the three biomarkers were found in both groups (r=0.402/P<0.001; r=0.724/P<0.001), with HIF-1α correlating with body mass index (BMI) in healthy controls (r=0.204/P=0.032). ROC analysis demonstrated high sensitivity and specificity in differentiating between groups (P<0.001), with cf-HbF showing the highest performance (AUC=0.923), followed by HIF-1α (AUC=0.882) and EPO (AUC=0.826). In conclusion, HIF-1α, EPO, and cf-HbF were associated with PFMCs and showed promising potential as biomarkers for distinguishing healthy from complicated pregnancies, with cf-HbF being the most significant. Their high levels also pointed to hypoxic adaptation dysfunction and/or resistance, as a pathogenic culprit.

## Introduction

In contrast to the physiological importance of hypoxia in the 1st trimester of pregnancy for critical stages of placental development, normal fetal organogenesis and growth are highly dependent on the extent of cellular normoxia and the tissue adaptive responses to hypoxia during the 2nd and 3rd trimester. Tissue oxygenation status controls the proliferation and differentiation of trophoblast stem cells and progenitor cells and the recruitment, phenotype, and function of maternal immune cells. Causes of hypoxia could be pre-placental, utero-placental, and/or fetal post-placental. Failure of the adaptive vascular and metabolic responses to intrauterine hypoxia is responsible for the persistent feto-maternal immediate and long-term consequences - the propensity for growth restriction, teratogenesis, stillbirth, preeclampsia, prematurity, and mortality (1-4).

The heterodimeric transcription factor hypoxia-inducible factor-1αβ (HIF-1αβ) is the dominant homeostatic regulator of cellular and developmental response to hypoxemia. Under normal oxygen conditions, inducible HIF-1α is quickly degraded through prolyl or asparaginyl hydroxylation, ubiquitination and proteasomal degradation, and interference with its binding to various co-activators (such as p300/CBP) (5). In the hypoxic milieu, these degradation mechanisms are inhibited, allowing HIF-1α to activate over 60 genes that enhance blood and oxygen supply, energy metabolism, and cell survival and regeneration. HIF-1-dependent biological activities include the angiogenic vascular endothelial growth factors (VEGFs), embryonic hemopoiesis and vascularization, embryonic placenta development, erythropoietin (EPO), and hormone biosynthetic and metabolic enzymes (5,6). HIF-1α can be stimulated independently of oxygen levels by inflammatory cytokines and NF-κB, nitric oxide, bacterial lipopolysaccharide, growth factors, and a wide range of infections. While hyperbaric oxygen (HBO_2_) boosts HIF-1α, peroxynitrite destabilizes HIF-1α. HBO_2_, HIF-1α imitators such as cobalt chloride, inhibitors of prolyl hydroxylases (PHs), iron (a cofactor for PHs) chelators such as desferrioxamine, and vHL antagonists, through stabilization of HIF-1α, are being tested in preclinical and clinical tests (7). Hypoxic stimuli following infarction involve HIF-1α, VEGF, TGF‐β, and soluble Fms‐like tyrosine kinase‐1 pathways. In the context of infection, HIF-1α enhances immune responses, including phagocytosis and bactericidal activity. It is mainly expressed in the hypoxic environment of early pregnancy, decreasing around the ninth week as oxygen levels rise (8,9). HIF-1α is linked to preeclampsia, with various microRNAs regulating its expression in chronic hypoxia associated with conditions like fetal growth restriction (10).

EPO, a glycoprotein cytokine, is a key regulator of erythropoiesis, primarily produced by adult kidneys, placenta, and fetal liver. EPO receptor (EpoR) activation on erythroid progenitor cells in the bone marrow enhances their survival, proliferation, and differentiation pathways through JAK2-STAT5 signaling, synergized by other growth factors (8,9). Hypoxia- and HIF-1α-dependent, the basal, very low level of EPO is increased to ∼1000-fold. Beyond its erythropoietic role, EPO exhibits various protective effects, including antioxidant, anti-inflammatory, antidiabetic, angiogenic and angio-protective, nephron-protective, hepatocyte regenerative, wound-healing, cardio-protective, and neurogenic and neuroprotective properties, and induces survival and mitosis of endothelial and vascular smooth muscle cells (11). During pregnancy, EPO levels increase 2-4 times, plateauing after 20 weeks to enhance blood flow and oxygen delivery. EPO and HIF-1α induce the expression of the angiogenic placental growth factor (12). Increased EPO levels in the amniotic fluid during fetal hypoxia in diabetic and Rh-immunized and preeclamptic pregnancies correlate negatively with cord blood pH and predict neonatal complications. Elevated fetal EPO indicates chronic intrauterine hypoxia, widespread fetal tissue damage, and metabolic dysfunction, which are implicated in the pathogenesis of macrosomia, diabetic pregnancy, preeclampsia, intrauterine growth restriction, small-for-gestational age babies, prolonged pregnancy, fetal anemia, meconium staining, fetal hemorrhage, abnormal fetal heart rate, and abnormal Doppler flow patterns (8,13).

Fetal hemoglobin (HbF; α2γ2) has a high affinity for oxygen, effectively extracting it from the maternal blood for fetal use. However, cell-free hemoglobin (cf-Hb), methemoglobin, and free heme and iron can cause oxidative damage to cells and tissues (14). HbF levels in amniotic fluid rise with gestational age, particularly in cases of preterm labor or premature membrane rupture (15). In preeclampsia, the placenta produces excess HbF, and the release of cf-HbF contributes to the blood-placental barrier inflammation and endothelial injury. Elevated cf-HbF in maternal circulation serves as an early biomarker for preeclampsia, indicating depletion of hemoglobin-scavenging proteins, namely hemopexin, heme oxygenase 1, and haptoglobin in the first trimester (16). Increased maternal circulating HbF and cf-HbF, from either fetal or maternal sources, negatively affect fetal and placental health by promoting mitochondrial dysfunction, oxidative stress, and inflammation and impairing angiogenesis and vascular integrity. These mechanisms are associated with preeclampsia, fetal growth restriction, and stillbirth (17-19).

The pathogenesis of most at-risk pregnancies (high-risk pregnancies and their offspring) is under-investigated, particularly the role of noninvasive biomarkers of hypoxia. Therefore, we cross-sectionally investigated changes in maternal plasma levels of HIF-1α, EPO, and cf-HbF in correlation with observed feto-maternal pregnancy outcomes and assessed their biomarker potential.

## Material and Methods

### Setting and participants

This cross-sectional study was conducted at Aljouf Maternity and Children Hospital in Sakaka, Saudi Arabia, beginning January 1 and ending December 12, 2022. The study protocol received approval from the Bioethical Committee of Jouf University (#6-16-4/40). Written informed consent was obtained from all participants. Exclusion criteria included uncertain diagnosis, obesity, immobilization, use of anti-convulsant or anti-inflammatory drugs, acute infections, malabsorption syndromes, anemia and hemoglobinopathies, endocrine disorders (hypo-/hyper-parathyroidism, thyroid disorders, and diabetes other than gestational diabetes mellitus), chronic comorbid medical conditions (e.g., kidney or liver impairment, and inflammatory and immunological disorders), superimposed preeclampsia, multiple pregnancies, and antepartum hemorrhage.

### Examination and investigations

Out of 450 pregnant women attending the hospital during the data collection period, 254 volunteered and met the inclusion criteria. Participants were divided into two groups: a control group of 136 women with normal feto-maternal outcomes and a complicated pregnancy group of 118 participants. The complicated group was further stratified by type of feto-maternal complication: severe preeclamptic toxemia with intrauterine growth restriction (PET-IUGR; n=30), gestational diabetes mellitus (GDM; n=53), abortion (n=14), undisturbed ectopic pregnancy (ETP; n=8), and inevitable preterm labor with premature rupture of membranes (PTL+PROMs; n=13). Both control and complicated participants were stratified by pregnancy duration.

After collecting the medical history (age, gravidity, parity, pregnancy duration, and significant medical history) and conducting a general examination (vital signs and weight and height for body mass index (BMI) in kg/m^2^), a systematic physical examination was performed. Pregnancy was assessed ultrasonographically for viability, fetal biometry, amniotic fluid condition, and fetal anomalies. Women with routine laboratory findings contradicting the inclusion criteria were excluded.

Five mL of peripheral venous blood was collected aseptically in EDTA tubes to recover plasma via centrifugation (2000 *g* for 5 min at 10°C), and aliquots were frozen at -80°C until batch analysis. HIF-1α (pg/mL), cf-HbF (ng/mL), and EPO (pg/mL=x 0.119 mIU/mL) were quantitatively measured in triplicates using specific sandwich ELISA assays (Sunlong Biotech Co. Ltd., China; cat# SL0905, SL2902Hu, and SL0679Hu). Control samples were spiked with the standard analyte and recovery being 99.8±8.4, 97.2±7.8, and 102.3±9.9% for sensitivity limit of 4.5 pg/mL, 1 ng/mL, and 0.7 pg/mL, respectively, and intra-assay CV of <10% for all of them.

### Statistical analysis

Data were analyzed using SPSS (version 23.0; IBM Corp., USA). As EPO has a log-normal distribution, its content is reported in log values. Normally distributed variables are reported as means±SD, with comparisons made using one-way ANOVA and Bonferroni *post hoc* tests. Non-normally distributed variables are reported as median and interquartile range, using the Kruskal-Wallis test, with *post hoc* pairwise comparisons conducted using the Mann-Whitney U test. Receiver operating characteristic (ROC) curve analysis was utilized to determine the area under the curve (AUC) for HIF-1α, cf-HbF, and EPO to assess their sensitivity and specificity in differentiating cases from controls. Binary logistic regression analysis identified risk factors for complications related to the studied biomarkers. A P-value <0.05 at a 95% confidence level was considered significant.

## Results

### Characteristics of the participating pregnant women

The difference in mean maternal age (in years) between groups was non-significant (P=0.095). Mean maternal BMI (kg/m^2^) was significantly different (P=0.002) between groups, with women with GDM having higher BMI than the other groups, except those with PET-IUGR. Median gravidity was significantly different (P=0.013) between groups, with women with GDM having higher gravidity than those with ETP. Median parity was significantly different (P=0.011) between groups with GDM women having higher parity than those with ETP. Median pregnancy duration (in weeks) at the time of testing was significantly different between groups (P<0.001), with PTL-PROM women having longer duration than controls and women with PET-IUGR, followed by women with GDM, women with abortion, and women with PTL+PROM (5>1 and 2, 2>3>4>5) (Supplementary Table S1).

### Plasma levels of hypoxia biomarkers among pregnant women

Mean plasma HIF-1α levels (pg/mL) were significantly different among different study groups (P<0.001), with levels in healthy controls being lower than in women with GDM and PTL+PROM. Mean plasma EPO levels (pg/mL) among healthy controls and women with PET-IUGR were significantly lower than that among women with GDM and PTL+PROM. Mean plasma cf-HbF levels (ng/mL) among healthy controls were significantly lower than that in women with GDM, abortion, ETP, and PTL+PROM. Mean cf-HbF levels among women with PTL+PROM were significantly higher than those with PET-IUGR, GDM, abortion, and ETP (Supplementary Table S2).

The three biomarkers had a strong positive correlation in healthy controls and in women with complicated pregnancy (r=0.402/P<0.001 and r=0.724/P<0.001, respectively). EPO correlated positively with pregnancy duration (r=0.230 and P=0.015) and HIF-1α correlated positively with BMI (r=0.204 and P=0.032) in healthy controls only. The three biomarkers did not significantly correlate with pregnancy duration among women with complicated pregnancy, except for a positive relationship for EPO in women with ETP (r=0.913 and P<0.004).

### ROC and AUC analysis

**Figure 1:**
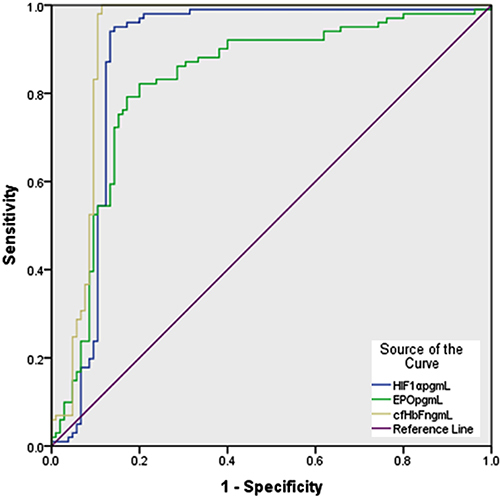
Receiver operating characteristic (ROC) curve for prediction of the occurrence of complications during pregnancy using plasma levels of hypoxia-inducible factor-1α (HIF-1α; pg/mL), erythropoietin (EPO; pg/mL, expressed in log values), and cell-free hemoglobin (cf-HbF; ng/mL) as hypoxia biomarkers in Saudi Arabian pregnant women.

Figure 1 and Table 1 show the ROC curve and AUC analysis, respectively. The three hypoxia biomarkers were significant predictors of complicated pregnancy. Their AUC values were close to 1, which shows that the model had a good fit. The AUC, sensitivity, and specificity were found to be highest for cf-HbF, thus proving it to be the best predictor.

**Table 1:** Mean area under the ROC curve (AUC) for differentiation between Saudi Arabian women with complicated pregnancies (n=118) and those with healthy pregnancies (n=136) using variation in plasma levels of hypoxia-inducible factor-1α (HIF-1α; pg/mL), erythropoietin (EPO; pg/mL; log values), and cell-free hemoglobin (cf-HbF; ng/mL) as hypoxia biomarkers.

Variable	Mean AUC±SEM	P	Asymptotic 95%CI		Cut-off value
			Lower Bound	Upper Bound	
HIF-1α	0.882±0.029	<0.001	0.825	0.939	≥94.24
Log EPO	0.828±0.031	<0.001	0.768	0.888	≥1.608
cf-HbF	0.923±0.023	<0.001	0.877	0.969	≥49.59

Data are reported as mean±SEM.

### Relationships among pregnant women's characteristics and the investigated hypoxia biomarkers

A logistic regression was performed to assess the effects of age, BMI, gravidity, parity, pregnancy duration, and the three hypoxia biomarkers on the likelihood of adverse pregnancy outcomes. The model explained 64.1% (P<0.001) of the variation and correctly classified 84.5% of the cases. Increasing levels of cf-HbF or HIF-1α was found to be associated with an increased likelihood of adverse pregnancy outcomes (Table 2).

**Table 2:** Multivariable binary logistic regression analysis of the relationship of each of the characteristics of the studied Saudi Arabian pregnant women and the investigated hypoxia biomarkers with adverse pregnancy outcomes.

Variables in the equation	Beta	S. E.	Wald	df	P value	OR	95%CI for OR	
							Lower	Upper
Step 1^a^								
Age	0.027	0.059	0.207	1	0.649	1.027	0.914	1.154
BMI	0.145	0.160	0.823	1	0.364	1.156	0.845	1.580
Gravidity	0.274	0.246	1.240	1	0.265	1.315	0.812	2.129
Parity	-0.426	0.312	1.871	1	0.171	0.653	0.355	1.203
PD	-0.024	0.019	1.504	1	0.220	0.977	0.940	1.014
HIF-1α	-0.043	0.009	23.299	1	0.000	0.958	0.941	0.975
EPO	-0.009	0.010	0.874	1	0.350	0.991	0.971	1.010
cf-HbF	0.164	0.026	40.097	1	0.000	1.178	1.120	1.239

^a^Variables entered in Step 1 were age, BMI, gravidity, and parity. PD: pregnancy duration in weeks; HIF-1α: hypoxia-inducible factor-1α; EPO: erythropoietin; cf-HbF: cell-free hemoglobin.

## Discussion

This study evaluated the changes in maternal circulating levels of HIF-1α, EPO, and cf-HbF as biomarkers of hypoxia in relation to feto-maternal pregnancy outcomes. The findings suggest that these pathogenic biomarkers have significant clinical utility, as their elevated levels correlate with adverse pregnancy outcomes, indicating a failure in hypoxic adaptation.

Women experiencing preterm labor and premature rupture of membranes had the highest plasma HIF-1α levels, followed by those with GDM, abortion, and ectopic pregnancy, while the lowest levels were found in preeclampsia, where differences were not significant. HIF-1α emerged as a strong predictive biomarker, closely linked to adverse outcomes, which is crucial given that 5-8% of pregnancies are affected by preeclampsia, a major contributor to maternal and fetal morbidity. Despite extensive research into preeclampsia's pathophysiology, effective preventive measures remain elusive. Early biomarkers are essential for timely interventions. HIF-1α plays a critical role in hypoxic conditions associated with preeclampsia, contributing to dysangiogenesis (20-22). Preeclamptic placentas have a 50% reduction in utero-placental circulation and frequent infarcts (23). Additionally, high altitudes have been shown to exacerbate preeclampsia through increased HIF-1α target gene expression (TGF-β3 in the placenta and EPO and VEGF in the mothers' blood), suggesting a link between environmental factors and disease prevalence (24). HIF-1α was considered to be the molecular link between preeclampsia and intrauterine growth retardation, downstream mediators (e.g., endothelin-1), and placental hypoxia (25,26). However, missed abortion with redox imbalance reduces HIF-1α expression (27). Increases in hypoxic systemic markers (HIF-1α, EPO, and VEGF) and proteasomal dysfunction correlate with lower birthweight-to-placental weight ratios and intrauterine growth restriction (24,28,29). Negative correlations were found between serum concentrations of each HIF-1α, hepcidin, and IL-6 and the week of delivery, and are promising biomarkers for the differentiation of PTL and term delivery (30). On the contrary, reduction in HIF-1α protein is implicated in preeclampsia (31).

EPO levels were also highest in women with PTL and PROMs, followed by those with GDM, abortion, and ETP, with the lowest levels recorded for preeclampsia. EPO demonstrated a strong predictive capacity, albeit weaker than HIF-1α and cf-HbF. Previous reports showed that bleeding-complicated pregnancy together with plural pregnancies have been associated with higher serum EPO values (13,32). Fetal hyperglycemia and hyper-insulinemia independently cause fetal hypoxia. Fetal hypoxia in diabetic pregnancy increases EPO levels in amniotic fluid, which correlate with maternal HbA1c levels. Hb glycosylation reduces oxygen delivery to tissues owing to the increased Hb-O_2_ affinity (1,33). Elevated EPO concentrations are often linked to impaired placental perfusion and hypoxemia, leading to increased production by the placenta and kidneys (34). Interestingly, previous studies report that preeclamptic patients do not show significant increases in serum EPO compared to healthy controls. While EPO has beneficial effects on metabolism, further research is needed to determine its clinical implications in diabetic pregnancies. Additionally, maternal obesity significantly contributes to GDM and adversely affects pregnancy outcomes. On the contrary, other reports stated that preeclamptic patients had insignificant elevation in serum EPO compared to healthy patients (35,36). Placental dysfunction-induced fetal hypoxia is accompanied by increased amniotic EPO levels and myocardial and brain injuries in 91% of cases of structurally normal stillbirth fetuses (37). Although preeclampsia is a hypoxic setting with induced EPO, paradoxically, there is reduced VEGF and increased levels of antiangiogenic factors (38). This supports our assumption of hypoxic adaptation dysfunction and/or resistance. Reportedly and similar to our findings, serum EPO levels are markedly increased in women with abnormal placentation (preeclampsia, pregnancy-induced hypertension, and IUGR) compared with healthy controls and positively correlated with uterine artery pulsatility index (39). Compared to healthy non-diabetic pregnancies, pregestational diabetic pregnancies have 2-4 times higher neonatal mortality and 4-6 times higher stillbirth rates, owing to fetal hypoxia with marked increases in amniotic fluid EPO levels that correlates with maternal HbA1c levels (40).

Women with PTL and PROMs exhibited significantly higher levels of cf-HbF compared to abortion, ectopic pregnancy, GDM, and preeclampsia, which had the lowest level. cf-HbF was the most powerful predictive biomarker among the three studied, correlating with high risks of adverse outcomes. Elevated cf-HbF levels in preeclampsia may lead to placental oxidative damage, feto-maternal barrier leakage, and compromised maternal vascular integrity, e.g., glomerular endotheliosis - a pathognomonic kidney damage for preeclampsia (41,42). Moreover, the increased release of placental syncytio-trophoblast-derived extracellular vesicles is associated with hypertension severity (43). The preeclamptic placenta increased HbF and released cf-HbF is a pathogenic factor for placental and other tissue damage in the first trimester (15-19,41-44). cf-Hb impairs vascular function and blood flow by sequestering NO·, being proinflammatory (activates NF-κB pathway and induces secretion of IL-1α and TNF-α) and increasing vascular resistance associated with fetal growth restriction. The inability to handle the elevated fetal heme load is expressed as high cf-HbF in pregnancies with fetal growth restriction and stillbirth (18). Furthermore, women with high HbF levels are more prone to deliver growth-restricted or small for gestational age fetuses. Preeclampsia is associated with increased HbF-concentrations and overwhelmed physiological Hb heme scavenger systems, despite their overactivation. Elevated levels of plasma HbF and α1-microglobulin (the extravascular Hb, heme, and radical scavenger) were observed in women with preeclampsia, presenting a potential predictive role for HbF (44,45).

Massive inter-individual variations in the three biomarkers were noticed among PET-IUGR patients that nullified the significance of their clear mean difference from the control. This could be due to the variability in the severity and pregnancy age at presentation of the cases - on top of the limited number of patients per group. Inter-individual differences were milder within the other patient groups that showed significantly higher content compared to healthy pregnancy - with slight differences amongst them except for PTL+PROM. The latter could be related to the acute nature of the cases and the stress to which both the mother and the fetus are subjected in such cases, not only from hypoxia but also from the increased stress of losing the baby. A similar scenario could also explain the performance of the biomarkers in the abortion cases. With the lowest number per group, the shorter pregnancy age recorded for ETP cases that lies within the anaerobic period of the 1st trimester could explain the weaker changes observed in them.

### Limitations

While our study offers valuable insights, the limited types of feto-maternal outcomes and small sample size in each subgroup may hinder the generalizability of the results. Many families in our community with high-risk pregnancies may seek care at larger facilities out of town, limiting participant diversity.

## Conclusions

Despite the robustness of our findings, future studies should be longitudinal, involve larger cohorts, and be multicentric to establish these biomarkers as standard diagnostic, pathogenic, and therapeutic targets, with defined norms and cut-off values.

## Supplementary Material

Click here to view [pdf].
