# Supplementary material for: Correlation of changes in circulating levels of hypoxia-induced factor-1α, erythropoietin, and cell-free fetal hemoglobin with feto-maternal pregnancy outcomes
Source: Braz J Med Biol Res. 2025 Oct 6;58:e14354. doi: 10.1590/1414-431X2025e14354 (PMC12513700; doi:10.1590/1414-431X2025e14354)
Supplement: Supplementary file 1 [file 1414-431X-bjmbr-58-e14354-suppl.pdf]

Table S1. Intergroup comparison of maternal age (years), body mass index (BMI; kg/m^2), and gravidity, parity, and pregnancy duration (PD; weeks) for Saudi Arabian women with healthy pregnancies (HCs) *vs* complicated pregnancies.

Group (G)	n	Age; mean±SD (Median, P25, P75)	P	BMI; mean±SD (Median, P25, P75)	P	Gravidity; median (P25, P75)	P Ph-PC	Parity; median (P25, P75)	P Ph-PC	PD; median (P25, P75)	P Ph-PC
HCs (G1)	136	29.618 ± 5.916 (30, 25, 34)	0.095*	22.30 ± 1.87 (22.6, 21.3, 23.65)	0.002* G3>G1, G4, G5, and G6	4, 2, 6	0.013[#] G3>G5	2, 1, 4	0.011[#] G3>G5	33, 19.5, 38	<0.001[#] G6>G1 and 2, G2>G3>G4>G5
PET-IUGR (G2)	30	31.500 ± 5.158 (31.5, 29, 35)		22.78 ± 1.70 (23.5, 21.8, 24.2		4, 2, 5		2, 1, 4		32.5, 28, 35	
GDM (G3)	53	31.189 ± 5.677 (31, 26, 34)		23.35 ± 1.81 (24.1, 22.6, 24.7)		5, 3, 6		3, 2, 4		23, 14, 32	
Abortion (G4)	14	31.071 ± 5.00 (31.5, 26, 34)		22.16 ± 2.16 (22.25, 20.8, 24.2)		3.5, 3, 5		2, 1, 3		10, 8, 16	
ETP (G5)	8	27.125 ± 6.490 (26, 21.5, 30.5)		20.88 ± 2.43 (21.05, 19, 21.95)		2.5, 2, 4.5		1, 0, 2.5		6, 6, 7	
PTL+PROM (G6)	13	27.769 ± 4.604 (27.769 ± 4.604)		21.87 ± 2.30 (21.4, 19.8, 24.2)		3, 1, 7		2, 0, 4		35, 34, 39	

Preeclampsia (PET-IUGR); gestational diabetes (GDM); ectopic pregnancy (ETP); preterm labor (PTL) and premature rupture of membranes (PROM; PTL+PROM). Data are reported as n, mean±SD (median, P25, P75). *One-way ANOVA test and Bonferroni *post hoc* pairwise comparisons (Ph-PC); [#]Kruskal-Wallis test and Ph-PC with Mann-Whitney U test.

Table S2. Plasma levels of the hypoxia biomarkers hypoxia-inducible factor-1α (HIF-1α; pg/mL expressed in log values), erythropoietin (EPO; pg/mL expressed in LOG values), and cell-free fetal hemoglobin (cf-HbF; ng/mL) among Saudi Arabian women with healthy pregnancies (HCs) *vs* those with complicated pregnancies.

Group (G)	n	HIF-1α; mean±SD (median, P25, P75)	P Ph-PC	Log EPO; median (P25, P75)	P Ph-PC	cf-HbF; mean±SD (median, P25, P75)	P Ph-PC
HCs (G1)	130	92.792 ± 82.521 (70.5, 62.6, 79.8)	<0.001 G1/G2=0.086 G1/G3<0.001 G1/G4=0.362 G1/G6=0.017 All other comparisons=0.999.	1.5, 1.5, 1.6	<0.001 G1/G3<0.001 G1/G4=0.184 G1/G6<0.001 G2/G3=0.010 G2/G6=0.036 All other comparisons=0.999.	42.598 ± 26.108 (35.9, 32.1, 40.9)	<0.001 G1/G2=0.074 G1/G3<0.001 G1/G4=0.002 G1/G5=0.046 G1/G6<0.001 G2/G6<0.001 G3/G6=0.002 G4/G6=0.029 G5/G6=0.09 All other comparisons=0.999.
PET-IUGR (G2)	30	124.103 ± 21.09 (118.7, 111.5, 133.0)		1.6, 1.5, 1.7		66.566 ± 16.974 (61.1, 54.0, 72.0)	
GDM (G3)	51	151.405 ± 77.299 (132.1, 114.4, 152.4)		1.8, 1.7, 2.0		84.750 ± 46.007 (70.2, 64.0, 81.2)	
Abortion (G4)	13	146.276 ± 64.005 (131.4, 123.9, 133.9)		1.8, 1.7, 1.8		87.927 ± 38.184 (79.2, 67.5, 93.8)	
ETP (G5)	8	127.929 ± 12.546 (130.1, 119.6, 134.2)		1.8, 1.7, 1.8		86.901 ± 8.870 (87.2, 79.7, 93.9)	
PTL+PROM (G6)	13	170.379 ± 165.582 (121.6, 113, 128.0)		1.8, 1.7, 1.8		139.038 ± 123.480 (85.0, 81.3, 99.5)	

Preeclampsia (PET-IUGR); gestational diabetes (GDM); ectopic pregnancy (ETP); preterm labor (PTL) and premature rupture of membranes (PROM; PTL+PROM). Data are reported as n, mean±SD, and median, P25, P75. P-values of one-way ANOVA and Bonferroni *post hoc* pairwise comparisons (Ph-PC).
